# The Influence of a Sudden Increase in Playing Time on Playing-Related Musculoskeletal Complaints in High-Level Amateur Musicians in a Longitudinal Cohort Study

**DOI:** 10.1371/journal.pone.0163472

**Published:** 2016-09-22

**Authors:** Laura M. Kok, Saskia Haitjema, Karlijn A. Groenewegen, A. Boni M. Rietveld

**Affiliations:** 1 Department of Orthopedics, NoordWest Ziekenhuis Groep, Alkmaar, The Netherlands; 2 Medical Center for Dancers and Musicians, Medisch Centrum Haaglanden, The Hague, The Netherlands; 3 Julius Centre for Health Sciences and Primary Care, University Medical Center Utrecht, Utrecht, The Netherlands; 4 Department of Orthopedics, Leiden University Medical Center, Leiden, The Netherlands; Universidad de Salamanca, SPAIN

## Abstract

**Background:**

Several studies in the domain of professional musicians describe the relation between playing time and the occurrence of musculoskeletal complaints in professional musicians. To date, no longitudinal cohort study into this relationship has been performed and no amateur musicians were studied. Therefore, the aim of this study is to examine the causal relationship between a sudden increase in playing time among amateur musicians on the occurrence of musculoskeletal complaints in a prospective cohort study.

**Methods:**

All members of two national Dutch Students Orchestras were asked to participate in the study. These project-based orchestras, consisting of high-level amateurs, followed a nine-hour rehearsing schedule for ten consecutive days. On the first day (t_0_) and after one week (t_1_) the subjects were asked to complete a paper-based questionnaire including sociodemographic characteristics, music-related questions, questions regarding playing-related musculoskeletal complaints and the music module of the disabilities of arm, shoulder and hand questionnaire.

**Results:**

The NSO consisted of 85 and the NESKO of 41 members during the study period. 59 subjects completed the questionnaire at both timepoints (response rate 47%). 9 subjects were excluded for being a music academy student, leaving 50 subjects (mean age 22.1, 72% female) suitable for analysis. During the rehearsal week, the prevalence of at least one playing-related musculoskeletal complaint increased from 28% to 80%. The most frequently affected areas were the neck, upper and lower back, hand/and or wrists and shoulders. The DASH music module score increased from 14 at t_0_ to 23 at t_1_.

**Conclusion:**

A point prevalence of 28% at the start of the study that increased remarkably to 80% within a one-week period. Future research should evaluate other risk factors for musculoskeletal complaints in amateur musicians. These risk factors should be the base for the development of preventive measures.

## Introduction

Nearly 20% of the Dutch population considers itself amateur musician[1], and in the USA there are over 62 million active amateur musicians.[2] Whereas the knowledge of health problems among professional musicians is growing[3], little is known about the health effect of playing a music instrument on an amateur level.[4] For example, the prevalence rates of musculoskeletal complaints among children and adolescents, music academy students and professional musicians are increasingly studied[5], whereas amateur musicians seem to be underrepresented. This is remarkable as most musicians are amateur musicians and therefore a possible health problem in this group is clearly relevant in terms of public health.

Several studies in the domain of professional musicians describe the association between playing time and the occurrence of musculoskeletal complaints. These studies, all with a cross-sectional design, report conflicting results. Ackermann et al [cite: MPPA 2012] evaluated professional orchestra musicians with musculoskeletal complaints.[6] These musicians self-reported insufficient rest (81%), long practice sessions (82%) and a sudden increase in playing time (76%) as causative factors for their complaints. In another study among professional orchestra musicians a positive correlation was found between the average of playing hours in an orchestra and playing-related musculoskeletal complaints.[7] Conversely, in a cross-sectional study among piano teachers, playing time was inversely related with musculoskeletal complaints.[8]

To date, no longitudinal cohort study has been performed among amateur musicians. Therefore, the aim of this study is to examine the causal association between a sudden increase in playing time among amateur musicians on the occurrence of musculoskeletal complaints in a prospective cohort study.

## Methods

### Study design and subjects

We conducted a prospective cohort study, in which all members of the Dutch Students Orchestra (Nederlands Studenten Orkest; NSO) and Dutch student chamber orchestra (NEderlands Studenten Kamerorkest; NESKO) were invited to participate. The NSO and NESKO are project-based student orchestras, consisting of high level amateur musicians. Both orchestras hold an audition to select the best amateur student players from all over the Netherlands. Therefore passing this audition with good result is an inclusion criterium. Once a year, nine-hour rehearsals are scheduled on ten consecutive days, followed by one to two weeks of daily concerts. At the first day (t = 0) and after one week (t = 1) during the rehearsal period in February 2015 (NSO) and May 2015 (NESKO) the subjects were asked to complete a questionnaire. As our study focused on amateur musicians, participants attending professional musical education were excluded. The study protocol was approved by the regional ethical committee; (METC Zuid-West Holland, registration number 14–086). No consent was collected as data were analyzed anonymously.

### Questionnaire

The paper-based questionnaire included sociodemographic characteristics and music-related baseline questions. We asked participants for their gender, date of birth, weight, height, lifestyle habits (smoking, alcohol and exercise) and whether they were left- or right-handed. Then we asked them about their instrument and playing experience.

The part of the questionnaire focusing on playing-related musculoskeletal complaints was an adaptation of the Nordic Musculoskeletal Questionnaire (NMQ). The definition by Zaza of playing related musculoskeletal complaints was used[9]; ‘pain and other symptoms, that are chronic, beyond your control, and that interfere with the ability to play your instrument at the usual level’. Participants were asked if they had any complaints during the past week, and were subsequently asked to identify where these complaints were located using the body map of the NMQ. The following anatomic regions were distinguished: head, mouth/jaw, neck, upper back, lower back, shoulders (left and right), elbows (left and right), hands/wrists (left and right), hips/thighs (left and right), knees (left and right), feet/ankles (left and right). Finally to assess the impact on daily living, the music module of the DASH was included.[10]

### Data processing

All questionnaires were entered into a database, with a unique identifier for each questionnaire to keep the link between database and paper. All answers were entered into the database as close as possible to the information written down. If a range was given, this was changed to the lowest number during data-cleaning. Due to logistical constraints, we were unable to give each participant two questionnaires with identifiers at the start of the study. Therefore, the questionnaires from time 0 and time 1 were matched by a unique identifier comprising the participant’s orchestra and birth date. Therefore subjects who completed only one of the two questionnaires were excluded. Questionnaires with illegible or incomplete birthdates were also excluded.

### Data analysis

Baseline-variables were represented as medians and quartiles 1 and 3 for continuous variables and as a number with a percentage for categorical variables. For each complaint, proportion of those reporting it at time 0 and time 1 was calculated. Subsequently, the absolute and proportional increase or decrease was calculated. As we were dealing with paired data, McNemar’s test was used to test for significance in the difference in prevalence of complaints between time 0 and time 1. To compare the DASH-module between time 0 and time 1, we used a paired t-test. For all statistical tests, a two-sided p-value of < 0.05 was considered significant. All analyses were performed using R (version 3.2.2) in the RStudio environment (version 0.99.463)

## Results

The NSO consisted of 85 and the NESKO of 41 members during the study period. 59 subjects completed the questionnaire at both the first and second measuring moments, a response rate of 47%. 9 subjects were excluded for being a music academy students, leaving 50 subjects suitable for analysis.

**Fig 1:**
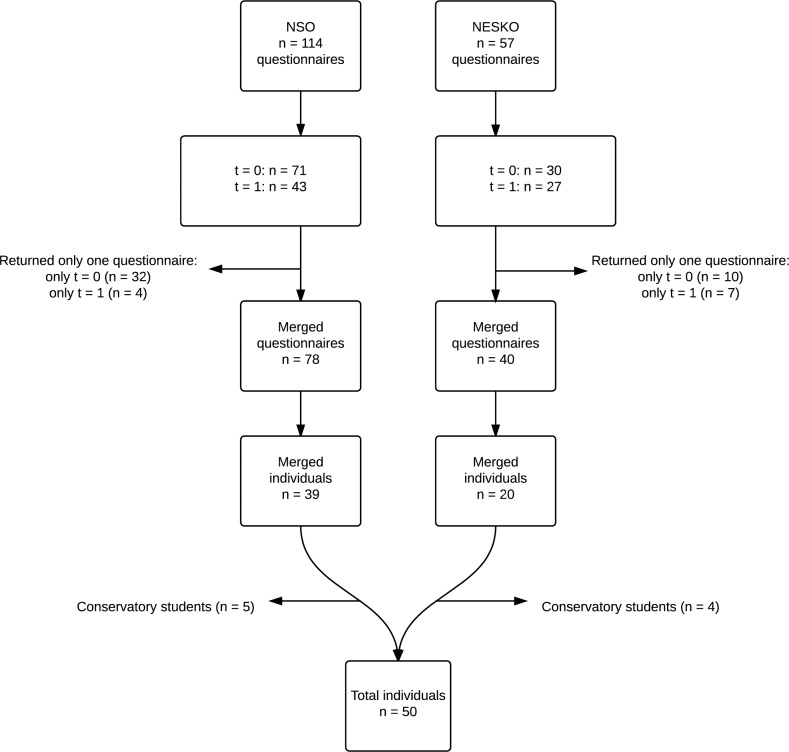
Flowchart. Flowchart of the inclusion process.

In Fig 1 a flowchart of the inclusion process is presented. The baseline characteristics of the included subjects are displayed in Table 1. The average age in the orchestras was 22.1 years (Q1-Q3 21.2–23.7), 72% of the study subjects were female. The orchestras consist of experienced amateur players, this is reflected in a median experience with the instrument of 13.5 years (Q1-Q3 11.2–15.0). The majority of players did warm-up before commencing rehearsals: 60%. The majority (84%) of respondents played a string instrument.

**Table 1:** Description of cohort.

Demographic information	Age (years)		22.1 (21.2–23.7)	
	Sex	Female	36 (72.00)	
		Male	14 (28.00)	
	Smoking	No	40 (80.00)	
		Yes	10 (20.00)	
Information related to playing	Exercise (hours/week)		2.0 (1.0–3.0)	
	Alcohol (units/week)		6.0 (3.0–10.0)	
	Warm-up duration (minutes)		10.0 (5.0–10.0)	
	Experience current instrument (years)		13.5 (11.2–15.0)	
	Dexterity	Right-handed	46 (92.00)	
		Left-handed	4 (8.00)	
	Warm-up	Yes	30 (60.00)	
		No	20 (40.00)	
Instrument	String-instrument	Total	43 (86.00)	
		Violin		24 (55.84)
		Viola		8 (18.60)
		Cello		8 (18.60)
		Double bass		3 (6.98)
	Wind-instrument	Total	5 (10.00)	
		Trumpet		1 (20.00)
		Horn		2 (40.00)
		Oboe		1 (20.00)
		Flute		1 (20.00)
	Percussion	Total	2 (4.00)	

Numbers represent median (Q1-Q3) for continuous variables and n (%) for categorical variables

### Playing related musculoskeletal complaints

At the start of the rehearsal period (t = 0) 28% of the musicians experienced at least one playing-related musculoskeletal complaint. At t = 1 this percentage had increased to 80%. There was one individual reporting complaints at the start of the study, but not after one week. Of those reporting no complaints at t = 0 (n = 36), 27 (75%) developed at least one complaint.

The reported playing-related musculoskeletal complaints at specific locations at t = 0 and t = 1 are displayed in Table 2. The most frequently affected areas are the neck, back and shoulders.

**Table 2:** Complaints in the last week at t0 and t1.

	T0	T1	Difference	P-value
No complaint	17 (34.0%)	9 (18.0%)	-8 (-16.0%)	0.080
Head	1 (2.0%)	4 (8.0%)	+3 (+6.0%)	0.371
Mouth/Jaw	2 (4.0%)	4 (8.0%)	+2 (+4.0%)	0.617
Neck	6 (12.0%)	29 (58.0%)	+23 (+46.0%)	<0.001
Upper back	7 (14.0%)	23 (46.0%)	+16 (+32.0%)	<0.001
Lower back	4 (8.0%)	16 (32.0%)	+12 (+24.0%)	0.001
Shoulders	9 (18.0%)	31 (62.0%)	+22 (+44.0%)	<0.001
Shoulder (left)	9 (18.0%)	29 (58.0%)	+20 (+40.0%)	<0.001
Shoulder (right)	5 (10.0%)	19 (38.0%)	+14 (+28.0%)	<0.001
Elbows	0 (0.0%)	5 (10.0%)	+5 (+10.0%)	0.074
Elbow (left)	0 (0.0%)	4 (8.0%)	+4 (+8.0%)	0.134
Elbow (right)	0 (0.0%)	2 (4.0%)	+2 (+4.0%)	0.480
Hands/wrists	3 (6.0%)	21 (42.0%)	+18 (+36.0%)	<0.001
Hand/wrist (left)	2 (4.0%)	10 (20.0%)	+8 (+16.0%)	0.027
Hand/wrist (right)	1 (2.0%)	13 (26.0%)	+12 (+24.0%)	0.001
Hips/upper legs	0 (0.0%)	2 (4.0%)	+2 (+4.0%)	0.480
Hip/upper leg (left)	0 (0.0%)	2 (4.0%)	+2 (+4.0%)	0.480
Hip/upper leg (right)	0 (0.0%)	1 (2.0%)	+1 (+2.0%)	1.000
Knees	0 (0.0%)	1 (2.0%)	+1 (+2.0%)	1.000
Knee (left)	0 (0.0%)	1 (2.0%)	+1 (+2.0%)	1.000
Knee (right)	0 (0.0%)	0 (0.0%)	+0 (+0.0%)	NA
Feet/ankles	0 (0.0%)	0 (0.0%)	+0 (+0.0%)	NA
Foot/ankle (left)	0 (0.0%)	0 (0.0%)	+0 (+0.0%)	NA
Foot/ankle (right)	0 (0.0%)	0 (0.0%)	+0 (+0.0%)	NA

### DASH music module

The score of the DASH module ranges between 0 (best score) and 100 (worst score). The score of the DASH music module in our study population was 14 at t = 0, compared to 23 at t = 1 (p = <0.001).

## Discussion

We studied the effect of a sudden large increase in playing time on musculoskeletal complaints in high-level amateur classical musicians. In our study-population, the prevalence of playing-related musculoskeletal complaints was 28% at baseline. After one week of intensive rehearsals, this percentage had increased to 80%.

Probably the most obvious explanation for the sharp increase in reported complaints is the sudden increase in playing time. Amateurs, including high-level amateurs, are not used to playing for long hours during consecutive days. Although we did not study the exact causal factors, we hypothesize that amateur musicians possess less technical strategies to cope with a sudden increase in playing time compared to professional musicians. This is for example reflected by the fact that not all musicians in our population performed warming-up exercises. Our population further differs from professionals as during this natural experiment, players also experienced lack of sleep and (for some) higher than normal alcohol consumption. These might play a role as aggravating factors in the development of playing-related musculoskeletal complaints.[11]

Almost all complaints in our population were reported in the upper body, most notably the neck and shoulders. This distribution of localizations of complaints has been described before. In a systematic review of studies describing the occurrence of musculoskeletal complaints among professional musicians, neck, shoulders and back were the most prevalent complaints.[3]

The sudden increase in musculoskeletal complaints at a music camp has been described before in folk musicians, yet in a less extreme environment. Buckley and Manchester reported a point prevalence of 44% at the end of a music camp for amateur folk-instrumentalists, and an incidence of 31% of overuse injury during the camp.[12] Interestingly, as these folk instrumentalists played on average less hours a day (3.7 hours a day in the Buckley study vs 9 hours a day in our population) the increase in prevalence was also less extreme (25% increase in the Buckley study vs a 52% increase in our population). This contributes to the hypothesis that there is a gradual association between the amount of increase in playing burden and the resulting playing-related musculoskeletal complaints. It should be realized that the participants in this study already are a fine selection of high level amateur musicians. Therefore, the healthy worker effect is applicable to this study: subjects with serious musculoskeletal complaints are more likely to drop out before reaching the acquired level or will at least reconsider taking part in such an intensive project.

Despite the fact that these rehearsal weeks can be considered an extreme form of exercise, both due to the extensive duration of practice and due to the high level of performance aspired by the orchestras, this does not mean that these findings are only relevant for this very select population and therefore cannot be generalized. Although we studied two groups highly educated, Caucasian, classical musicians, many of them women, almost all musicians are amateurs. In addition almost all musical activities undertaken in a group show large variation in the amount of playing time as orchestras preparing for concerts plan extra rehearsal evenings and rehearsal time spikes during rehearsal weekends. Moreover, the consequences of developing a playing related musculoskeletal complaints are most likely not confined to playing the instrument, as the distribution of complaints points towards locations that are also frequently used in daily life (e.g. back pain while sitting in a chair, wrist pain with repetitive hand motions like typing). This could lead to loss of productivity at work. However, despite the influence of the complaints on playing capacity, it is unknown what the impact of these complaints on other daily activities.

This study however has some limitations. First of all, our definition of playing-related musculoskeletal complaints does not strictly exclude complaints that are not caused by playing the instrument. This could lead to information bias, where complaints of another cause were misclassified as playing-related musculoskeletal complaint. However, we did stress (both in the oral instructions and on the questionnaire itself) the definition of playing-related musculoskeletal complaints as stated by Zaza.[9] Furthermore, the prevalence of for lower extremity complaints was very low among our amateur musicians. This despite the fact that lower extremity complaints are more frequently reported compared to upper extremity complaints in the open population.[13]

Playing-related musculoskeletal complaints very probably do not have one causal factor, but are more likely a multifactorial issue. The effect of a sudden increase in playing time could be modified by other contributing factors like poor posture while playing, technique and lack of sleep. Above the ‘tour-life’, being away from home can influence the complaints. In addition, although part of the questionnaire we used was derived from the Nordic Musculoskeletal questionnaire, in its adapted form it was not revalidated. The DASH music module on the impact and significance of complaints while playing a musical instrument does not have norm scores for musicians. This should be taken into account while inteprating our results. When looking at the available information on this subpart of the DASH questionnaire, our t0 DASH score was somewhat higher compared to the general US population aged 19–34 (5.12)[14,15] As expected, the t1 DASH score in this study is more than one SD higher compared to the norm population. However, for the optional modules of the DASH the minimally clinically relevant differences are unknown. The clinical impact and of the above reported musculoskeletal complaints are supported by the fact that five musicians had to reduce their playing activity during the rehearsal period.

A second limitation is that while we aspired to have each participant fill out the questionnaire twice (once at study start and once at study end), this was not realized due to logistical constraints and people not handing in their questionnaire. We did have 47% participants with two questionnaires, however there were 42 subjects with only the first and 11 with only the second. However, we do not think the filling out the questionnaire only once was related to the presence of playing related musculoskeletal complaints. As such, it led to a decrease in our included sample size, but not to a (large) distortion of results.

Main strength of the study is the use of an existing event to study this scarcely studied population of classical amateur musicians. This gives the unique opportunity to study the association between a sudden increase in playing time and the development of playing-related musculoskeletal complaints and acquisition of a large sample size. We are the first to show that a sudden increase in playing time in high-level amateur classical musicians leads to complaints that inhibit an enjoyable leisure activity and can have implications on work in daily life.

Our study therefore stresses the need for implementing preventive measures for amateur musicians going on a music camp. Such preventive measures can take place both before and during the camp, for example by gradually increasing playing-time. Other possible preventive measures include advice on posture, and provision of quality furniture. Several studies show positive health outcomes as the result of an exercise and/or educational health program.[16–19] However, future studies should aim at improving dedicated preventive health progams for musicians. Optimal scientific quality should be pursued in these studies.Above, other risk factors for musculoskeletal complaints in amateur musicians should be evaluated in future research. These risk factors should be the base for the development of preventive measures.

## Supporting Information

S1 File Dataset. Full dataset of the current study.
(CSV)

S2 File Dutch questionnaire. Dutch version of the used questionnaire.
(DOCX)

S3 File English questionnaire. English version of the used questionnaire.
(DOCX)
