# Supplementary material for: The Influence of a Sudden Increase in Playing Time on Playing-Related Musculoskeletal Complaints in High-Level Amateur Musicians in a Longitudinal Cohort Study
Source: PLoS One. 2016 Sep 22;11(9):e0163472. doi: 10.1371/journal.pone.0163472 (PMC5033332; doi:10.1371/journal.pone.0163472)
Supplement: S2 File — Dutch version of the used questionnaire. (DOCX) [file pone.0163472.s002.docx]

**Vragenlijst MuZIEKonderZOEK Studienummer ………**

Algemene gegevens

1. Ik ben een vrouw/man *(doorhalen wat niet van toepassing is)*

2. Mijn geboortedatum is ……………………………

3. Ik ben ……… cm lang en weeg ……… kg

4. Ik rook wel/niet *(doorhalen wat niet van toepassing is)*

5. Ik ben links/rechtshandig *(doorhalen wat niet van toepassing is)*

6. Ik sport gemiddeld ……… uur in de week

7. Ik drink gemiddeld ……… glazen alcohol per week

Muziek-specifieke gegevens

8. Ik zit in het volgende orkest……………………………………………………………………………………

9. Ik bespeel in dit orkest het volgende muziekinstrument: ………………………………………

10. Ik bespeel dit instrument al ………… jaar

11. Ik heb wel/geen les op dit instrument *(doorhalen wat niet van toepassing is)*

12. Ik studeer …………. uur per week op dit instrument *(inclusief orkestrepetities)*

13. Speel je in (“warming-up”)?

- ja, namelijk ……… minuten
- nee

14. Ik ben wel/geen conservatoriumstudent *(doorhalen wat niet van toepassing is)*

Let op:

De volgende vraag gaat over speelgerelateerde klachten. Lees de vraag, de toelichting en het voorbeeld op de volgende bladzijde alsjeblieft aandachtig door.

15. Heb je wel eens last van speelgerelateerde klachten gehad?
*Onder deze klachten verstaan we pijn of andere klachten die langdurig zijn, die je niet onder controle hebt en die interfereren met je vermogen om je instrument te bespelen op je gebruikelijke niveau.*

- Ja, namelijk a. In de afgelopen week
  - - - Ja 🡪 vul poppetje 1 in en ga naar vraag b.
      - Nee 🡪 ga naar vraag b.

b. In de afgelopen vier weken

- Ja 🡪 vul poppetje 2 in en ga naar vraag c.
- Nee 🡪 ga naar vraag c.

c. In de afgelopen drie maanden

- Ja 🡪 vul poppetje 3 in en ga naar vraag d.
- Nee 🡪 ga naar vraag d.

d. In het afgelopen jaar

- Ja 🡪 vul poppetje 4 in
- Nee 🡪 ga naar vraag 16 (op de laatste bladzijde)
- Nee, nooit 🡪 ga naar vraag 16 (op de laatste bladzijde)

Voorbeeld: Pietje had een jaar geleden last van zijn linkerschouder, maar dat is nu over. Verder had hij een halfjaar tot drie maanden geleden last van zijn rechterhand. Afgelopen week heeft hij last gekregen van zijn linkerelleboog.
Pietje vult hierboven in:

a) Ja 🡪 en kruist het vakje elleboog links aan
b) Nee
c) Ja 🡪 en kruist het vakje hand rechts aan
d) Ja 🡪 en kruist de vakjes hand rechts en schouder links aan

NB: Als je de afgelopen week klachten hebt gehad, hoef je die klachten dus niet bij vier poppetjes in te vullen, maar alleen bij het eerste poppetje.

**Poppetje 1: Speelgerelateerde klachten afgelopen week**

Ik had klachten aan mijn:


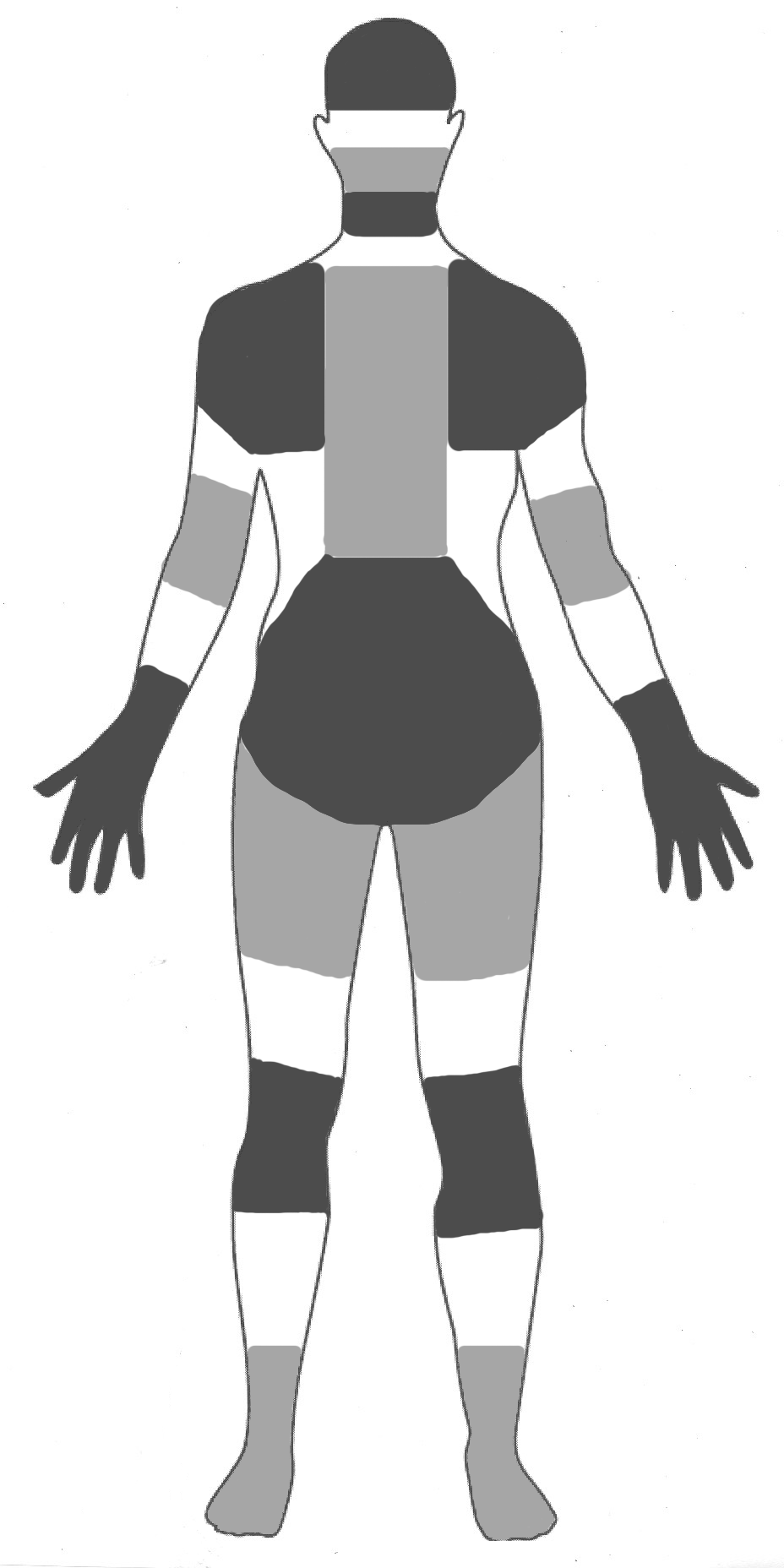


Links

Rechts

Hoofd

Mond/kaak

Nek

Schouder

Bovenrug

Elleboog

Onderrug

Hand/pols

Heup/
bovenbeen

Knie

Voet/enkel

- Hoofd
- Mond/kaak
- Nek
- Schouder links
- Schouder rechts
- Bovenrug
- Elleboog links
- Elleboog rechts
- Onderrug
- Hand/pols links
- Hand/pols rechts
- Heup/bovenbeen links
- Heup/bovenbeen rechts
- Knie links
- Knie rechts
- Voet/enkel links
- Voet/enkel rechts

**Poppetje 2: Speelgerelateerde klachten afgelopen vier weken**

Ik had klachten aan mijn:


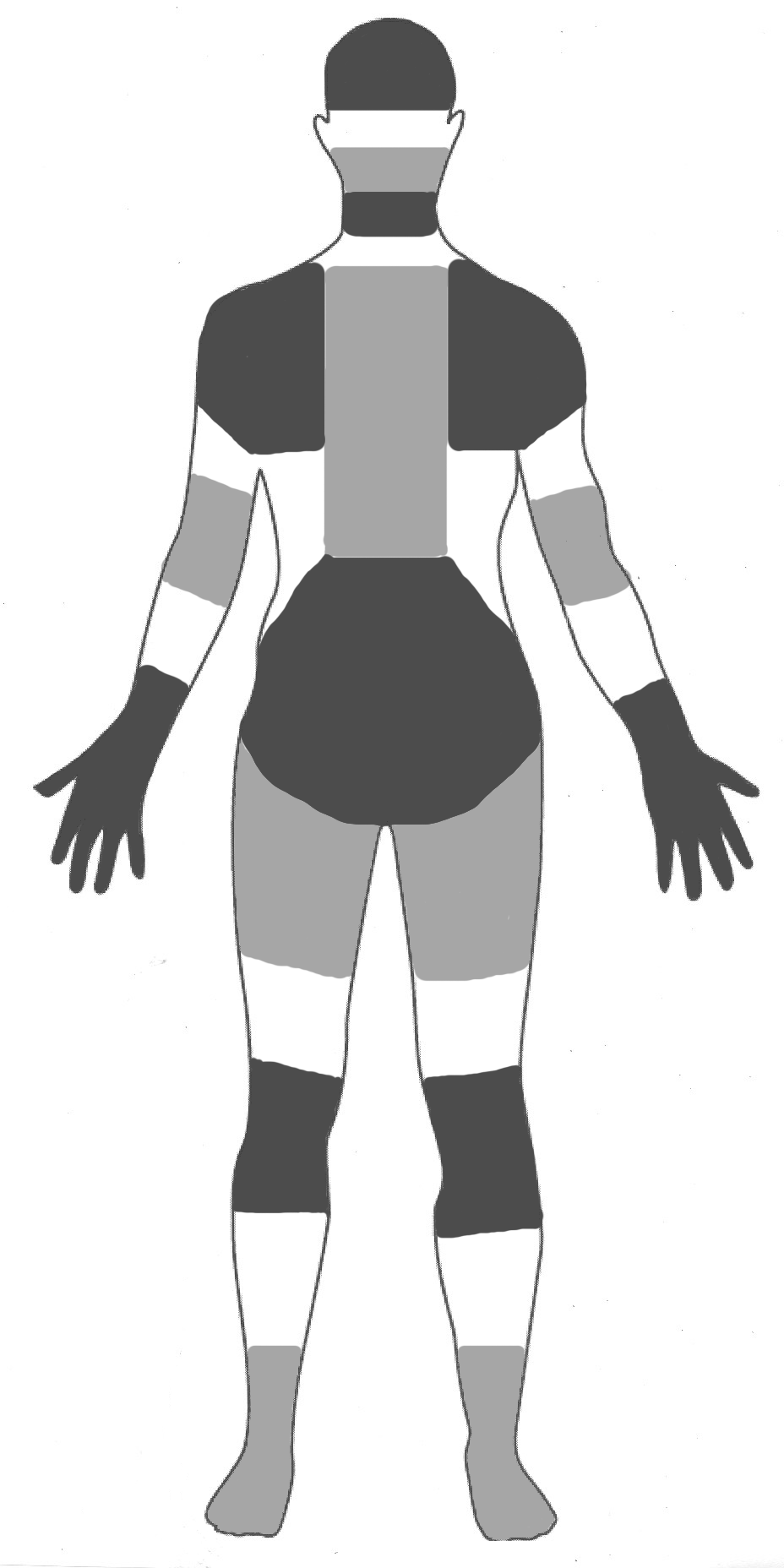


Links

Rechts

Hoofd

Mond/kaak

Nek

Schouder

Bovenrug

Elleboog

Onderrug

Hand/pols

Heup/
bovenbeen

Knie

Voet/enkel

- Hoofd
- Mond/kaak
- Nek
- Schouder links
- Schouder rechts
- Bovenrug
- Elleboog links
- Elleboog rechts
- Onderrug
- Hand/pols links
- Hand/pols rechts
- Heup/bovenbeen links
- Heup/bovenbeen rechts
- Knie links
- Knie rechts
- Voet/enkel links
- Voet/enkel rechts

**Poppetje 3: Speelgerelateerde klachten afgelopen drie maanden**

Ik had klachten aan mijn:

- Hoofd


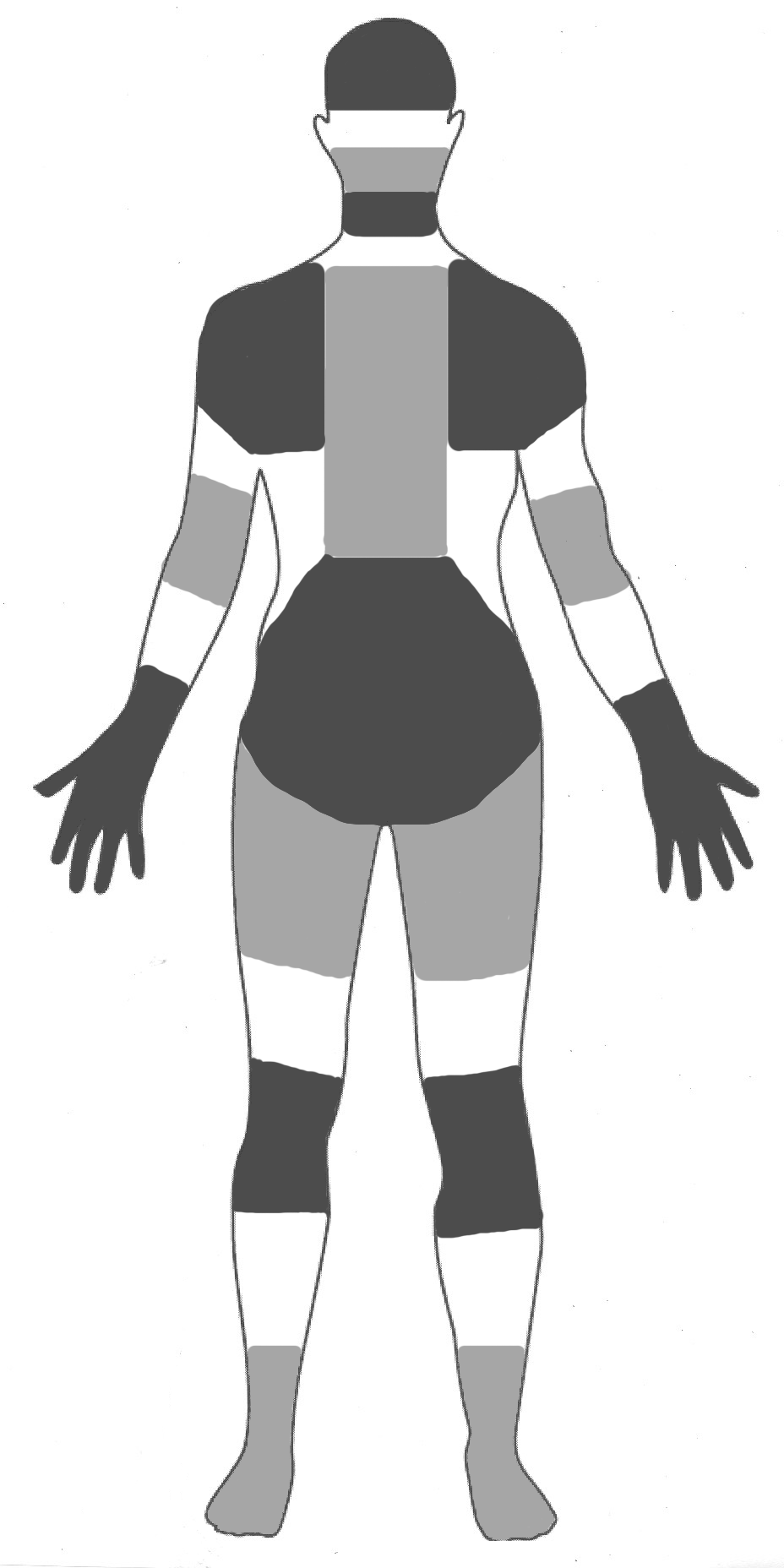


Links

Rechts

Hoofd

Mond/kaak

Nek

Schouder

Bovenrug

Elleboog

Onderrug

Hand/pols

Heup/
bovenbeen

Knie

Voet/enkel

- Mond/kaak
- Nek
- Schouder links
- Schouder rechts
- Bovenrug
- Elleboog links
- Elleboog rechts
- Onderrug
- Hand/pols links
- Hand/pols rechts
- Heup/bovenbeen links
- Heup/bovenbeen rechts
- Knie links
- Knie rechts
- Voet/enkel links
- Voet/enkel rechts

**Poppetje 4: Speelgerelateerde klachten afgelopen jaar**

Ik had klachten aan mijn:


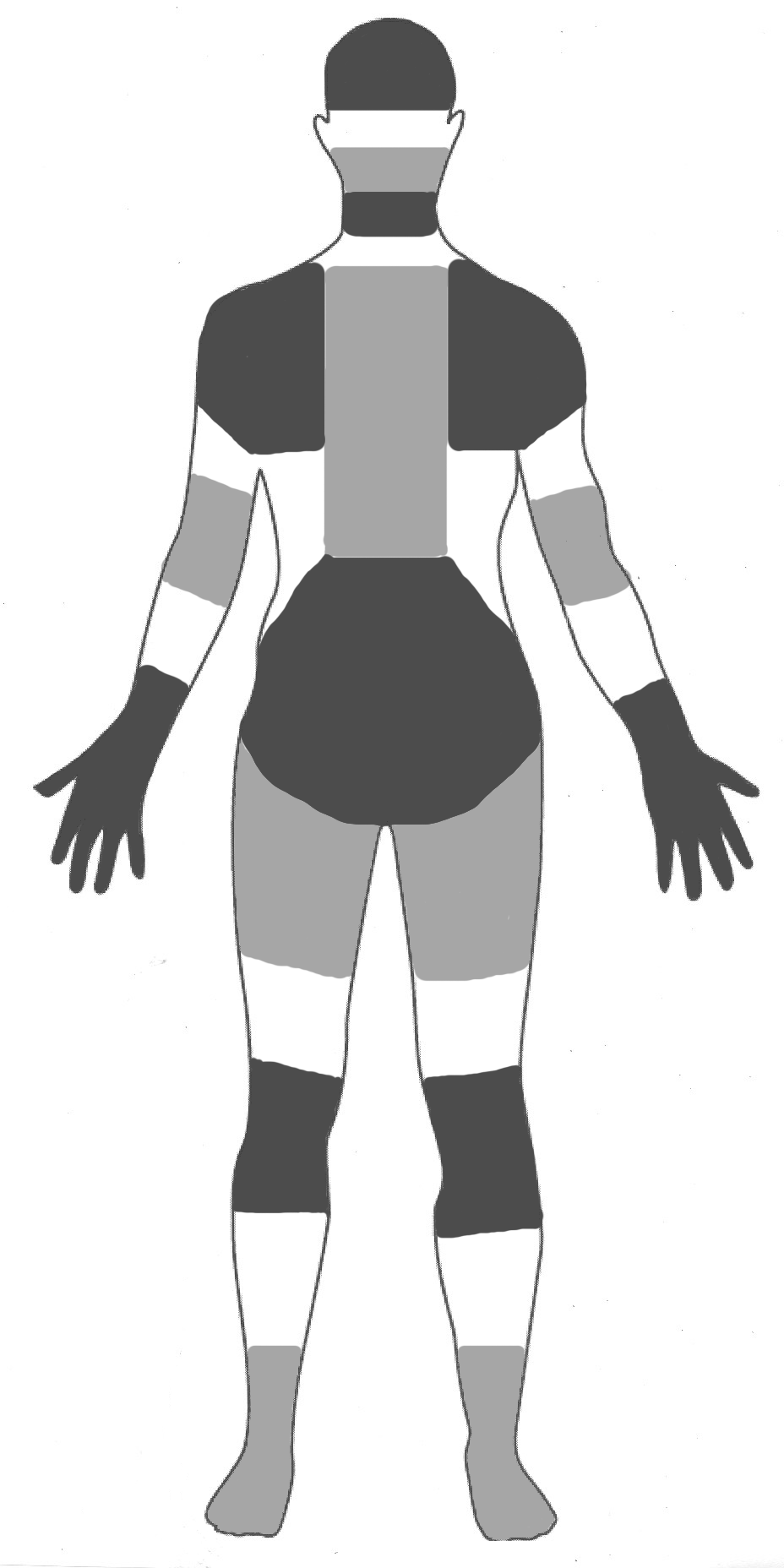


Links

Rechts

Hoofd

Mond/kaak

Nek

Schouder

Bovenrug

Elleboog

Onderrug

Hand/pols

Heup/
bovenbeen

Knie

Voet/enkel

- Hoofd
- Mond/kaak
- Nek
- Schouder links
- Schouder rechts
- Bovenrug
- Elleboog links
- Elleboog rechts
- Onderrug
- Hand/pols links
- Hand/pols rechts
- Heup/bovenbeen links
- Heup/bovenbeen rechts
- Knie links
- Knie rechts
- Voet/enkel links
- Voet/enkel rechts

16. Omcirkel het getal dat het beste je lichamelijke mogelijkheden beschrijft
***in de afgelopen week.***

Had je de afgelopen week problemen met:

|  | Geen probleem | Gering probleem | Probleem | Ernstig probleem | Niet mogelijk |
| --- | --- | --- | --- | --- | --- |
| Het gebruiken van normale technieken voor het bespelen van je instrument? | 1 | 2 | 3 | 4 | 5 |
| Het bespelen van je instrument ten gevolge van arm, schouder of handpijn? | 1 | 2 | 3 | 4 | 5 |
| Het bespelen van je instrument zo goed als je zou willen? | 1 | 2 | 3 | 4 | 5 |
| Het besteden van de gebruikelijke tijd aan het bespelen van je instrument? | 1 | 2 | 3 | 4 | 5 |

Dat was de laatste vraag. Dankjewel voor je medewerking!
