# Supplementary material for: The Influence of a Sudden Increase in Playing Time on Playing-Related Musculoskeletal Complaints in High-Level Amateur Musicians in a Longitudinal Cohort Study
Source: PLoS One. 2016 Sep 22;11(9):e0163472. doi: 10.1371/journal.pone.0163472 (PMC5033332; doi:10.1371/journal.pone.0163472)
Supplement: S3 File — English version of the used questionnaire. (DOCX) [file pone.0163472.s003.docx]

**Questionnaire MuZIEKonderZOEK Study number**

General information

1. I am a woman/man *(delete what is not appropriate)*

2. My date of birth is ……………………………

3. I am……… cm long en my weight is ……… kg

4. I am a smoker yes/no *(delete what is not appropriate)*

5. I am left/righthanded *(delete what is not appropriate)*

6. I sport on average ……… hours a week

7. I drink on average ……… glasses of alcohol a week

Music-specific information

8. The orchestra I am in is………………………..…………………………………………………………………

9. In this orchestra I play the following musical instrument: ……………………………………..

10. I have been playing this musical instrument for ………… years

11. I am taking lessons on this instrument yes/no *(delete what is not appropriate)*

12. I study …………. hours a week on this instrument *(including repetitions)*

13. Do you do a “warming-up” before playing?

- yes, namely ……… minutes
- no

14. I am a conservatory student yes/no *(delete what is not appropriate)*

Please pay attention:

The following question is about playing-related complaints. Please read the question, the explanation and the example on the next page carefully.

15. Have you ever had playing-related complaints?
*Playing-related complaints are pain and other symptoms that are chronic, beyond your control and that interfere with your ability to play your instrument at the usual level*

- Yes, namely a. In the past week
  - - - Yes 🡪 fill in body chart 1 in and go to question b.
      - No 🡪 go to question b.

b. In the past four weeks

- Yes 🡪 fill in body chart 2 in and go to question c.
- No 🡪 go to question c.

c. In the past three months

- Yes 🡪 fill in body chart 3 in and go to question d.
- No 🡪 go to question d.

d. In the past year

- Yes 🡪 fill in body chart 4
- No 🡪 go to question 16 (on the last page)
- No, never 🡪 go to question 16 (on the last page)

Example: Joe had shoulder complaints (left) a year ago, but that is gone now. Furthermore, he had from 6 till 3 months ago complaints of his right hand. Last week he had complaints of his left elbow.
Joe fills in:

a) Yes 🡪 and ticks the box next to left elbow
b) No
c) Yes 🡪 and ticks the box next to right hand
d) Yes 🡪 and ticks the boxes next to right hand and left shoulder

NB: So if you only had complaints in the past week, you don’t need to fill out all four body charts, but just the first one

**Body chart 1: Playing-related complaints past week**

I had complaints of my:


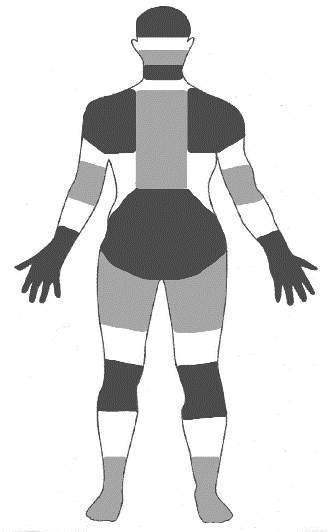


Left

Right

Head

Mouth/jaw

Neck

Shoulder

Upper back

Elbow

Lower back

Hand/wrist

Hip/
upper leg

Knee

Foot/ankle

- Head
- Mouth/jaw
- Neck
- Shoulder left
- Shoulder right
- Upper back
- Elbow left
- Elbow right
- Lower back
- Hand/wrist left
- Hand/wrist right
- Hip/upper leg left
- Hip/upper leg right
- Knee left
- Knee right
- Foot/ankle left
- Foot/ankle right

**Body chart 2: Playing-related complaints past four weeks**

I had complaints of my:


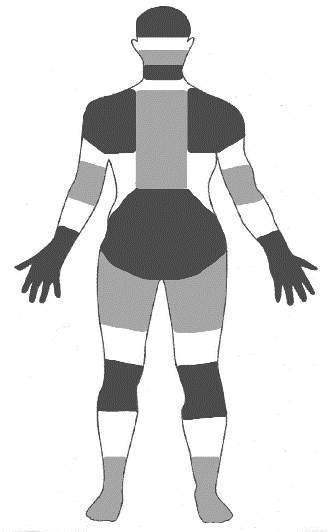


Links

Rechts

Head

Mouth/jaw

Neck

Shoulder

Upper back

Elbow

Lower back

Hand/wrist

Hip/
upper leg

Knee

Foot/ankle

- Head
- Mouth/jaw
- Neck
- Shoulder left
- Shoulder right
- Upper back
- Elbow left
- Elbow right
- Lower back
- Hand/wrist left
- Hand/wrist right
- Hip/upper leg left
- Hip/upper leg right
- Knee left
- Knee right
- Foot/ankle left
- Foot/ankle right

**Body chart 3: Playing-related complaints past three months**

I had complaints of my:

- Head


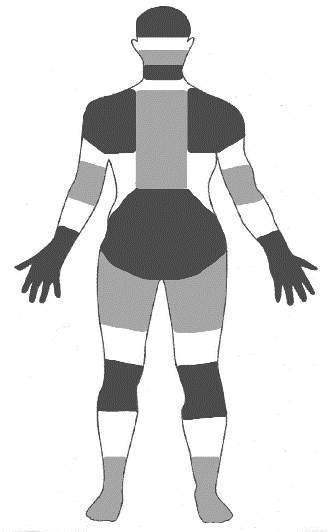


Links

Rechts

Head

Mouth/jaw

Neck

Shoulder

Upper back

Elbow

Lower back

Hand/wrist

Hip/
upper leg

Knee

Foot/ankle

- Mouth/jaw
- Neck
- Shoulder left
- Shoulder right
- Upper back
- Elbow left
- Elbow right
- Lower back
- Hand/wrist left
- Hand/wrist right
- Hip/upper leg left
- Hip/upper leg right
- Knee left
- Knee right
- Foot/ankle left
- Foot/ankle right

**Body chart 4: Playing-related complaints past year**

I had complaints of my:


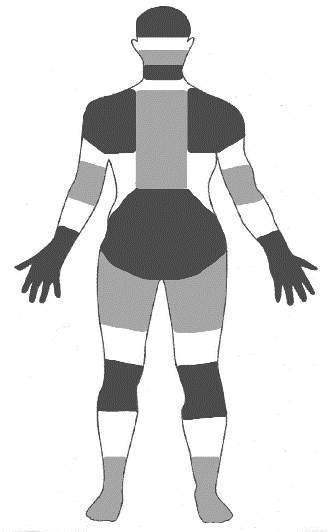


Links

Rechts

Head

Mouth/jaw

Neck

Shoulder

Upper back

Elbow

Lower back

Hand/wrist

Hip/
upper leg

Knee

Foot/ankle

- Head
- Mouth/jaw
- Neck
- Shoulder left
- Shoulder right
- Upper back
- Elbow left
- Elbow right
- Lower back
- Hand/wrist left
- Hand/wrist right
- Hip/upper leg left
- Hip/upper leg right
- Knee left
- Knee right
- Foot/ankle left
- Foot/ankle right

16. Please circle the number that best describe your physical ability
***during the past week.***

Did you have any difficulty:

|  | No  difficulty | Mild difficulty | Moderate difficulty | Severe difficulty | Unable |
| --- | --- | --- | --- | --- | --- |
| Using your usual technique for playing your instrument? | 1 | 2 | 3 | 4 | 5 |
| Playing your musical instrument because of arm, shoulder or hand pain? | 1 | 2 | 3 | 4 | 5 |
| Playing your musical instrument as well as you would like? | 1 | 2 | 3 | 4 | 5 |
| Spending your usual amount of time practising or playing your instrument? | 1 | 2 | 3 | 4 | 5 |

That was the last question. Thank you for your cooperation!
